# 4H-SiC LDMOS Integrating a Trench MOS Channel Diode for Improved Reverse Recovery Performance

**DOI:** 10.3390/mi14050950

**Published:** 2023-04-27

**Authors:** Yanjuan Liu, Dezhen Jia, Junpeng Fang

**Affiliations:** 1 College of Electronic and Information Engineering, Shenyang Aerospace University, Shenyang 110136, China; liuyanjuan@hrbeu.edu.cn (Y.L.);; 2 School of Integrated Circuits, Tsinghua University, Beijing 100084, China

**Keywords:** LDMOS, MOS channel diode, reverse recovery

## Abstract

In this paper, a 4H-SiC lateral gate MOSFET incorporating a trench MOS channel diode at the source side is explored to improve the reverse recovery characteristics. In addition, a 2D numerical simulator (ATLAS) is used to investigate the electrical characteristics of the devices. The investigational results have demonstrated that the peak reverse recovery current is reduced by 63.5%, the reverse recovery charge is reduced by 24.5%, and the reverse recovery energy loss is decreased by 25.8%, with extra complexity in the fabrication process.

## 1. Introduction

Recently, laterally-diffused metal-oxide semiconductor (LDMOS) devices have been widely applied for AC-DC power supply [1,2,3,4,5,6,7]. With the superior physical properties, such as a higher critical breakdown electric field, higher saturated electron drift velocity, and wider band gap, silicon carbide LDMOS devices have lower specific on-resistance compared with a silicon LDMOS under the same breakdown voltage [8,9,10,11,12]. In the AC-DC power supply, LDMOS needs to be anti-parallel to an external freewheeling diode in order to suppress the voltage surge. There exists a parasitic body diode, consisting of the P-body and N-drift region, which can be used as a freewheeling diode [13].

However, this cost- and area-saving alternative is not feasible in silicon carbide devices for the following two reasons: one is the high built-in potential of PN junction (close to 3 V), leading to a high conduction loss; the other is that the recombination of minority carriers can cause a bipolar degradation issue [14]. In order to realize this alternative, the reverse recovery performance needs to be improved. In order to overcome the abovementioned two issues (high conduction loss and bipolar degradation), that is to say, improving the reverse recovery performance of the parasitic body diode, many solutions have been utilized, such as the introduction of a Schottky contact [15,16,17] and integrating a channel diode [18,19,20,21] in the SiC VDMOSFET. Compared with SiC VDMOSFET, LDMOS is better for integration and is beneficial to the miniaturization of the AC-DC electric system. However, research into the electrical performance of the 4H-SiC LDMOS has few reports [22,23,24,25,26], but the reverse recovery performance has not been reported in the 4H-SiC LDMOS.

When the C-LDMOS device serves as a freewheeling diode, reverse conduction takes place through the parasitic P-body/N-drift junction. This parasitic P-body/N-drift junction is a bipolar device that could cause an inferior reverse recovery performance. In this paper, in order to improve the reverse recovery of C-LDMOS, the concept of the MOS channel diode is introduced to a 4H-SiC LDMOS device (called TMCD-LDMOS), which has been reported in other 4H-SiC switches [18,19,20]. Compared to the parasitic P-body/N-drift junction, the TMCD has the following effects when the device serves as a freewheeling diode. On the one hand, the trench MOS channel diode has a smaller turn-on voltage than the parasitic P-body/N-drift junction and the two diodes are in a series connection. When a LDMOS device works as a freewheeling diode, the TMCD diode turns on first, suppressing the turn-on of the parasitic body diode effectively. On the other hand, the TMCD diode is a unipolar device, which can reduce the injection of the minority carriers from the parasitic P-body/N-drift junction greatly and improve the reverse recovery performance.

In this paper, 4H-SiC LDMOS featuring a trench MOS channel diode is investigated in detail and compared with a conventional LDMOS. Note that due to the limitation of our present experimental conditions, we are unable to provide experimental results; however, simulation tools are generally used for the optimization and development of various semiconductor device structures in order to reduce the cost of device manufacturability and the development period and hence lower the risks associated with technology transfer in an industrial environment. Moreover, simulation tools are very useful to explore novel device architectural concepts for different material systems. So the aim of the simulation work is to compare the electrical characteristics of two different structures on the same terms while not revealing the physical devices’ features.

## 2. Setup of Simulation Conditions

**Figure 1:**
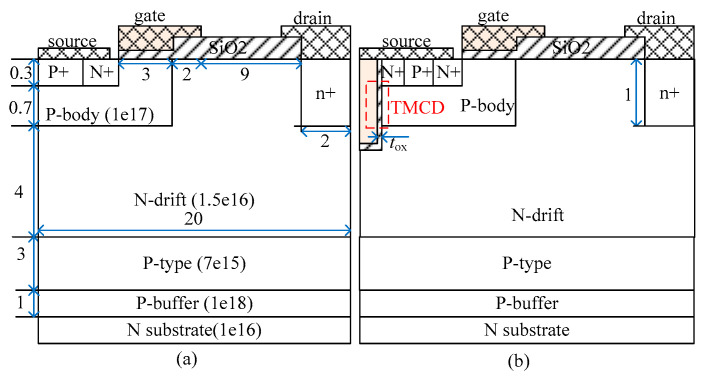
Cross-sectional schematic of half-cells of (**a**) C-LDMOS (**b**) TMCD-LDMOS.

The structures of the investigated C-LDMOS and TMCD-LDMOS device are shown in Figure 1, which is not to scale and the distances are in micrometers. C-LDMOS and TMCD-LDMOS have the same region parameters, except that a trench MOS channel diode exists in TMCD-LDMOS, which is shown within the dashed box in Figure 1b. The gate oxide thickness is 50 nm and the trench oxide thickness of TMCD is 30 nm. The doping concentration of N+, P+, and n+ regions are set as 1 × 10^19^ cm^−3^.

**Figure 2:**
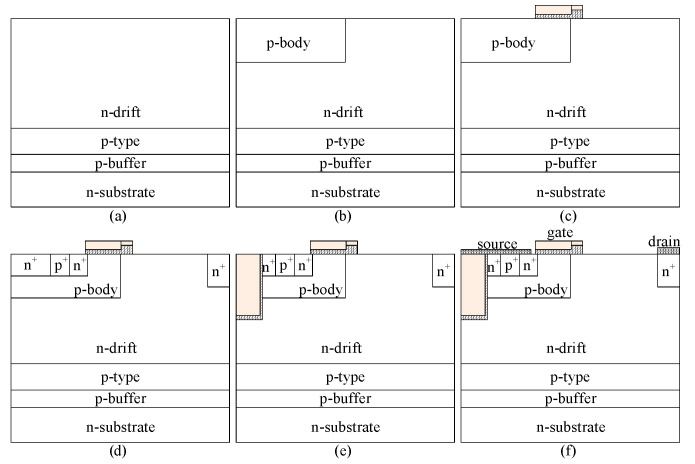
A possible fabrication process of TMCD-LDMOS.

A possible fabrication process for TMCD-LDMOS is shown in Figure 2. The key fabrication steps are as follows: (a) forming the p-buffer, p-type, and n-drift regions on the n+ wafer by epitaxial growth technology; (b) forming the p-body layer by ion implantation and performing the ion implantation annealing; (c) forming the gate oxide by thermal growth, depositing and polishing polysilicon; (d) forming the n and p region by ion implantation; (e) forming the trench by etching; (f) depositing and polishing polysilicon, forming the gate, source and drain electrodes.

ATLAS, a 2D numerical simulator, is used to investigate and explore the device performances. The following simulation models are utilized: the bandgap narrowing model (BGN), which is important in heavily doped regions and critical for bipolar gain; AUGER, which describes the direct transition of three carriers and is important at high current densities; and Shockley–Read–Hall (SRH) for recombination and carrier lifetime models, which uses fixed minority carrier lifetimes, doping and temperature-dependent field mobility models (ANALYTIC) [27]; and Fermi Dirac statistics and Selberherr’s impact ionization model. The actual effects of the used models on the electrical performance can be found in Ref. [27]. The material parameters and mobility are set to the same as in TABLE I and TABLE II shown in reference [28]. In addition, the lifetime in the N-drift region is set as 1 μs.

## 3. Analysis and Discussions of Performances

In this part, the electrical performances are explored and compared between C-LDMOS and TMCD-LDMOS, which includes the static performances (forward I–V, BV, reverse conduction of the body diode) and the reverse recovery characteristic of the body diode.

### 3.1. Static Performances

**Figure 3:**
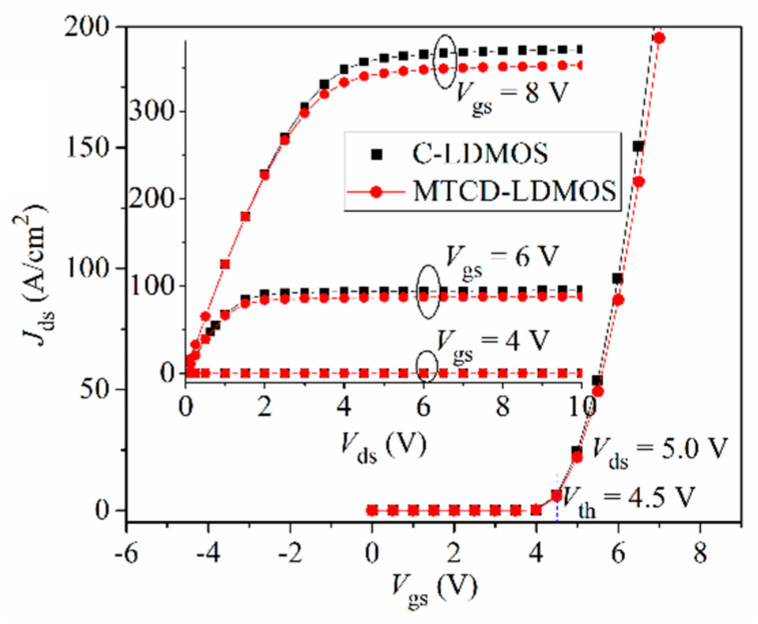
Comparison of output characteristics and transfer curves.

Figure 3 shows the current versus voltage characteristics of C-LDMOS and TMCD-LDMOS. From this figure, it can be seen that the introduction of TMCD has no effect on the specific on-resistance, which is 8.34 mΩ·cm^2^ at *V*_gs_ = 8 V and *J*_ds_ = 100 A/cm^2^ for C-LDMOS and TMCD-LDMOS. In addition, this figure presents the transfer characteristic at *V*_ds_ = 5 V, proving that the TMCD has no influence on the threshold voltage.

**Figure 4:**
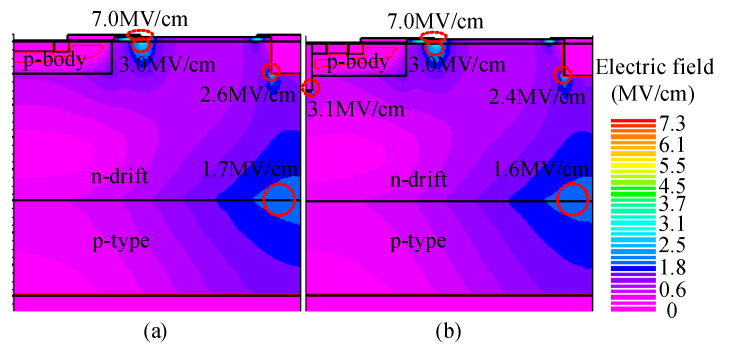
Electric field distribution for (**a**) C-LDMOS and (**b**) TMCD-LDMOS when *V*_gs_ = 0 V and *V*_ds_ = 1000 V.

The electric field characteristic is presented in Figure 4 when the devices are at avalanche multiplication. Obviously, the breakdown voltage is the same for the two structures due to the same parameters of the N-drift region and the TMCD is far from the blocking region of the drain voltage.

**Figure 5:**
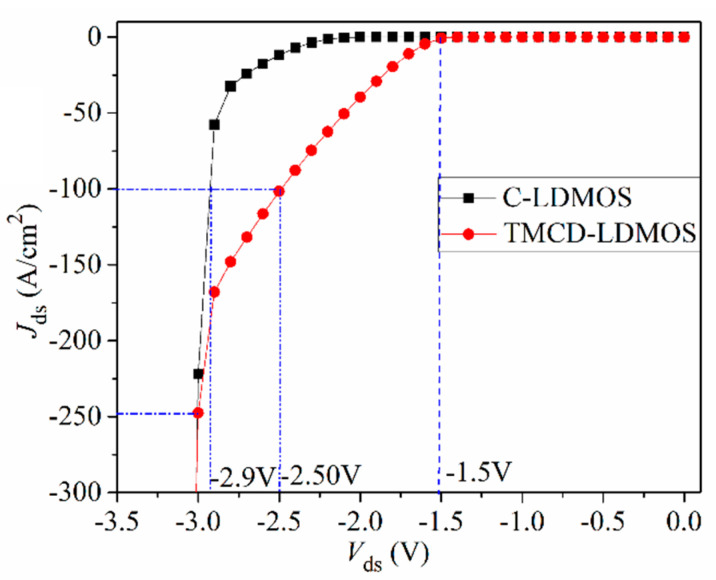
I–V performances of the parasitic body diode of the C-LDMOS and TMCD-LDMOS devices.

TMCD-LDMOS features a trench MOS channel diode, which has the following effects when the device serves as a freewheeling diode: on the one hand, it suppresses the turn-on of the PN junction diode effectively; however, it reduces the minority carrier concentration greatly, leading to a reduction in the power loss. Figure 5 shows the I–-V characteristics of the parasitic body diode of the two structures. From this graph, we can see that the parasitic body diode, composed of the PN junction body diode, turns on at about 2.3 V in the C-LDMOS. However, for the TMCD-LDMOS, the parasitic body diode is composed of the PN junction body diode and the trench MOS channel diode and the TMCD turns on at ~1.5 V first.

**Figure 6:**
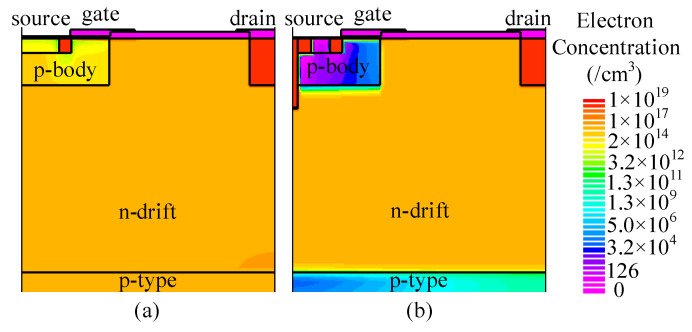
Electron concentration distribution of (**a**) C-LDMOS and (**b**) TMCD-LDMOS at *J*_ds_ = 100 A/cm^2^ when the device serves as a freewheeling diode.

**Figure 7:**
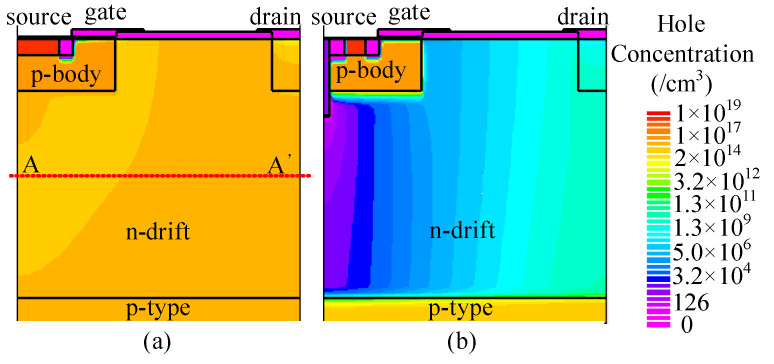
Hole concentration distribution of (**a**) C-LDMOS and (**b**) TMCD-LDMOS at *J*_ds_ = 100 A/cm^2^ when the device serves as a freewheeling diode.

**Figure 8:**
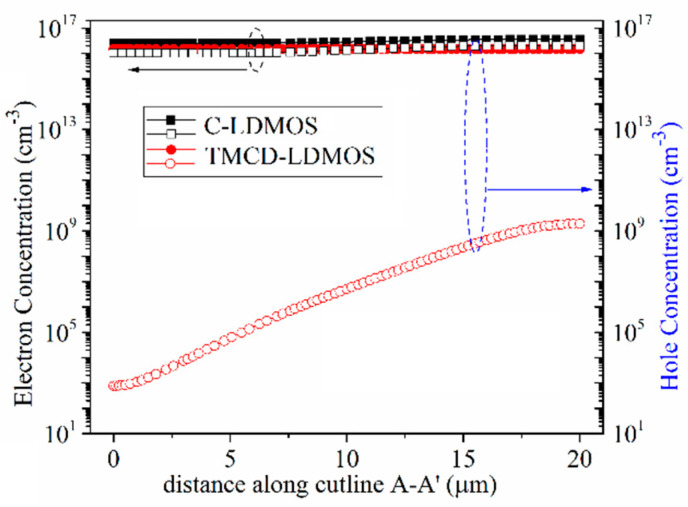
Carrier concentration distribution along cutline A-A’ (shown in Figure 7) at *J*_ds_ = 100 A/cm^2^ when the device serves as a freewheeling diode.

At *J*_ds_ = 100 A/cm^2^, the conduction voltage of the parasitic body diode is 2.9 and 2.5 V for C-LDMOS and TMCD-LDMOS, respectively. The trench MOS channel diode has a lower conduction voltage than a PN junction diode, suppressing the turn-on of the PN junction diode effectively. Moreover, the TMCD is a unipolar structure (electron-type), resulting in a lower injection of the minority carriers (hole carriers) in TMCD-LDMOS. The electron and hole concentration distributions are presented in Figure 6 and Figure 7 at *J*_ds_ = 100 A/cm^2^, proving that the TMCD-LDMOS has an extremely low hole concentration. The hole and electron concentration profiles along the cut-line A-A’ shown in Figure 7 are plotted in Figure 8, intuitively demonstrating the turn-on of the parasitic PN junction diode is effectively suppressed by the TMCD. Moreover, the reduction in the minority carriers can result in a superior reserve recovery characteristic and a lower reverse recovery energy loss, which is discussed in the next part, allowing the parasitic body diode to be used as a freewheeling diode.

### 3.2. Reverse Recovery Performance

When a MOSFET device works as a freewheeling diode, the reverse recovery performance of its parasitic body diode is very important and affects the system features. Thus, a MOSFET requires a superior reverse recovery characteristic when it is used as an AC-DC power supply.

The parasitic PN junction diode in C-LDMOS is a bipolar device and generates extensive hole carriers in the N-drift region working as a freewheeling diode. However, for the TMCD-LDMOS, a trench MOS channel diode is introduced, a unipolar device without an injection of the minority carrier when serving as a freewheeling diode. Moreover, the turn-on voltage of TMCD is lower and can suppress the turn-on of the parasitic PN junction diode. Thus, a LDMOS featuring a MOS channel diode can achieve a great improvement of the reverse recovery characteristic.

**Figure 9:**
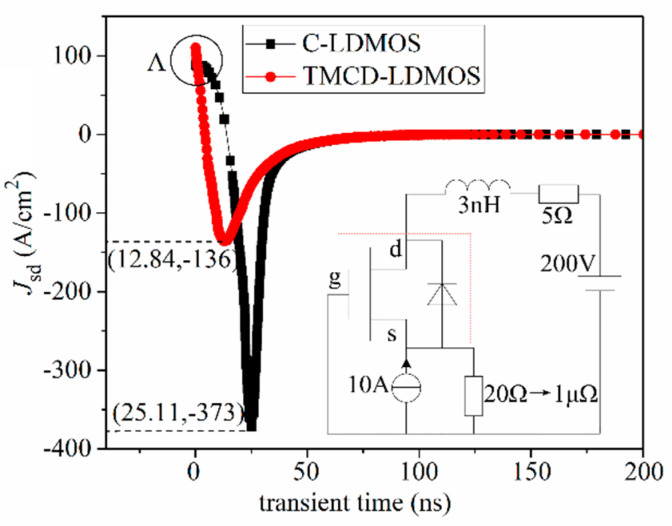
Comparison of the reverse recovery performances.

**Figure 10:**
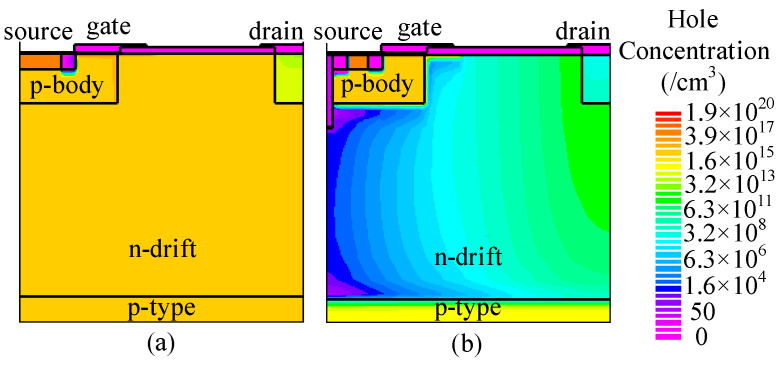
Hole concentration distribution of (**a**) C-LDMOS and (**b**) TMCD-LDMOS at point A shown in Figure 9 (*J*_sd_ = 100 A/cm^2^ when the device serves as a freewheeling diode).

As can be seen in Figure 5, the TMCD-LDMOS structure outperforms the conventional one for current density of <250 A/cm^2^ under this device’s parameters, and the current density can be changed by adjusting the parameters of the TMCD diode. So, in this paper, the current density of 100 A/cm^2^ is chosen to investigate the reverse recovery performance. The test circuit used for the reverse recovery performance and the simulation result is plotted in Figure 9 [29]. From this figure, it can be observed that the peak reverse recovery current density (*I*_RM_) is 136 and 373 A/cm^2^ for TMCD-LDMOS and C-LDMOS, respectively. TMCD-LDMOS has a 63.5% improvement in *I*_RM_ compared with C-LDMOS. Integrating current over time, the reverse recovery charge (*Q*_rr_) is 0.554 and 0.418 nC/cm^2^ for C-LDMOS and TMCD-LDMOS, respectively, in which *Q*_rr_ is reduced by 24.5% in TMCD-LDMOS. This proves that the trench MOS channel diode can achieve a smaller *Q*_rr_, which is beneficial for switching loss reduction. With the integration of the production of the drain voltage and source current with time, the reverse recovery energy loss (*E*_RR_) is 4.54 × 10^−5^ J/cm^2^ in TMCD-LDMOS, which is 25.8% lower than that of C-LDMOS (6.12 × 10^−5^ J/cm^2^). The hole carrier distribution at point A shown in Figure 9 is described in Figure 10, indicating that a lower hole carrier concentration exists in TMCD-LDMOS, which needs to be extracted during the reverse recovery process.

### 3.3. Parameter Influence

In this section, we investigate the effect of the tox of TMCD (shown in Figure 1) on the electrical characteristics, such as the I–V curve of the parasitic body diode and the reverse recovery performance.

**Figure 11:**
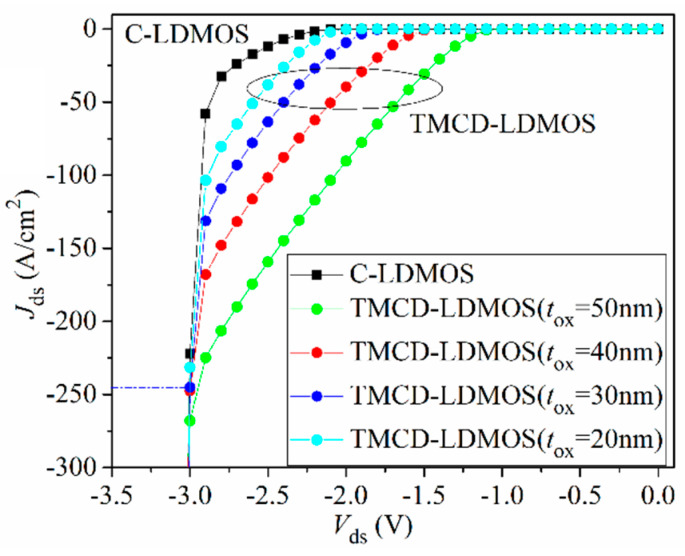
Influence of *t*_ox_ on reverse conduction performance.

**Figure 12:**
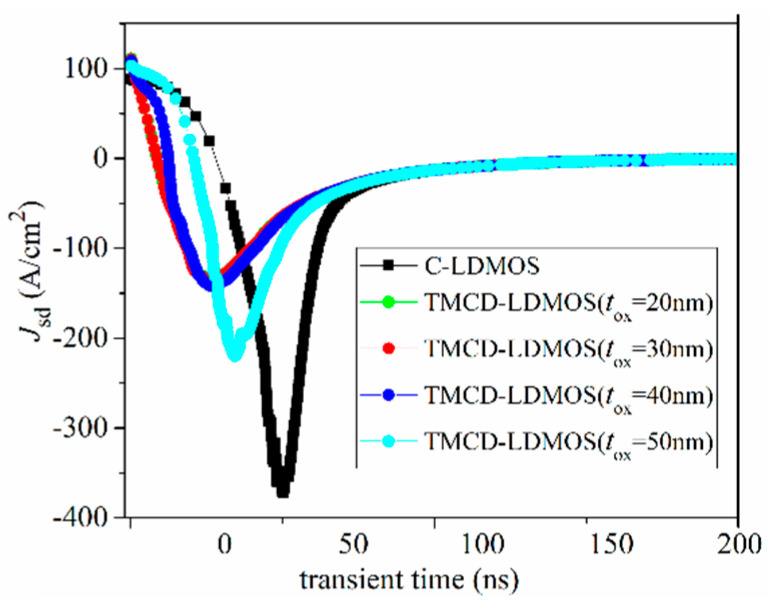
Influence of *t*_ox_ on reverse recovery performance.

Actually, the thickness of SiO_2_ in TMCD may not be equal to 30 nm, but varied around 30 nm, so it is necessary to explore the influence of *t*_ox_ on the characteristics of TMCD. Figure 11 presents the I–V curves of TMCD at different *t*_ox_. With decreasing *t*_ox_, the TMCD-LDMOS has a lower voltage drop and lower hole concentration under the same drain current when the device works as a freewheeling diode. This leads to the relationship between *t*_ox_ and reverse recovery curve presented in Figure 12. From Figure 11 and Figure 12, it can be determined that the TMCD-LDMOS has superior electrical characteristics when the *t*_ox_ changes due to the fabrication process technology. 

## 4. Conclusions

A 4H-SiC lateral MOSFET featuring a trench MOS channel diode at the source side is investigated in detail and compared with a conventional LDMOS. The drawback of the device is the extra complexity of the fabrication process. However, the research results have demonstrated that TMCD can effectively suppress the parasitic PN junction diode and significantly enhance the reverse recovery characteristic. Compared with C-LDMOS, TMCD-LDMOS has a 63.5% reduction in the peak reverse recovery current, a 24.5% reduction in the reverse recovery charge, and a 31.3% reduction in the reverse recovery energy loss, giving the device a significant advantage in high-frequency applications.

## Data Availability

Not applicable.
